# MOCVD Growth and Structural Properties of ZnS Nanowires: A Case Study of Polytypism

**DOI:** 10.3390/nano12142323

**Published:** 2022-07-06

**Authors:** Sumit Kumar, Frédéric Fossard, Gaelle Amiri, Jean-Michel Chauveau, Vincent Sallet

**Affiliations:** 1 Groupe d’Étude de la Matière Condensée (GEMAC), Centre National de la Recherche Scientifique, Université de Versailles St Quentin en Yvelines, Université Paris-Saclay, 45 Avenue des Etats-Unis, 78035 Versailles, France; sumit.kumar@uvsq.fr (S.K.); gaelle.amiri@uvsq.fr (G.A.); jean-michel.chauveau@uvsq.fr (J.-M.C.); 2 Laboratoire d’Étude des Microstructures (LEM), Centre National de la Recherche Scientifique, Office National d’Etudes et de Recherches Aérospatiales, Université Paris-Saclay, 29 Avenue Division Leclerc, 92322 Chatillon, France; frederic.fossard@onera.fr

**Keywords:** ZnS nanowires, MOCVD, polytypes, transmission electron microscopy

## Abstract

Controlling the morphology, orientation, and crystal phase of semiconductor nanowires is crucial for their future applications in nanodevices. In this work, zinc sulfide (ZnS) nanowires have been grown by metalorganic chemical vapor deposition (MOCVD), using gold or gold–gallium alloys as catalyst. At first, basic studies on MOCVD growth regimes (mass-transport, zinc- or sulfur- rich conditions) have been carried out for ZnS thin films. Subsequently, the growth of ZnS nanowires was investigated, as a function of key parameters such as substrate temperature, S/Zn ratio, physical state and composition of the catalyst droplet, and supersaturation. A detailed analysis of the structural properties by transmission electron microscopy (TEM) is given. Depending on the growth conditions, a variety of polytypes is observed: zinc-blende (3C), wurtzite (2H) as well as an uncommon 15R crystal phase. It is demonstrated that twinning superlattices, i.e., 3C structures with periodic twin defects, can be achieved by increasing the Ga concentration of the catalyst. These experimental results are discussed in the light of growth mechanisms reported for semiconductor nanowires. Hence, in this work, the control of ZnS nanowire structural properties appears as a case study for the better understanding of polytypism in semiconductor 1D nanostructures.

## 1. Introduction

The tremendous properties of semiconductor nanowires (SC NWs) open the field for advanced devices and new applications in electronics, optoelectronics, energy harvesting, batteries, sensors, or photocatalysis, *inter alia* [1,2,3,4,5,6,7]. Their fabrication is still the object of an intense research [8,9]. Among the possible nanostructured materials which are expected to enter our daily life, II-VI semiconductors have demonstrated their huge potential. For the last two decades, zinc oxide (ZnO) has been probably the most studied II-VI compound, as it is particularly suitable for its fabrication of the nanometer scale, giving rise to a variety of nano-particles, -rods, -belts, -tubes or -rings [10]. Selenide and telluride compounds, such as ZnTe, ZnSe, and CdSe, are promising building blocks for nanophotonics and light emission, including single-photon emission [11,12]. Also belonging to the II-VI family, zinc sulfide (ZnS) is an “old”, well-known semiconductor used for a long time as luminescent material, pigment, infrared windows, X-ray and neutron detectors. ZnS has a wide bandgap and commonly shows two polymorphs: zinc blende (ZB, cubic 3C, with Eg = 3.72 eV), and wurtzite (WZ, hexagonal 2H, with Eg = 3.77 eV). Actually, ZnS as bulk material, can form in a variety of crystallographic structures. This particular behavior is known as polytypism, and it is typical of other compounds such as SiC [13]. ZnS polytypes differ from each other only in the way Zn-S bilayers are stacked along the [111]—cubic or [1]—hexagonal directions. Mardix has reported a long list of crystal structures in Ref. [14], all currently identified, with a number of layers in the elementary stacking sequence up to 64. Interestingly, Engel et al. [15] have calculated the total energies using the first principle pseudopotential approach for 3C, 2H, 4H, 6H, and 6L polytypes, and found that all these energies are very close in ZnS. Other simple calculations from cohesive energies [16,17] lead to the same conclusion that different crystal phases should easily occur in this material. Indeed, this is in agreement with past experiments where 4H, 12R, and 15R polytypes have been clearly identified [18]. In this context, growing ZnS in the nanowire form is expected to further enhance the probability to create original polytypes. This is due to the unique and peculiar growth mechanism of SC NWs when a catalyst particle is used to trigger the emergence of the 1D nanostructure (leading to either wire, ribbon, belt, etc.). This catalyzed growth is called vapor-liquid-solid (VLS) or vapor-solid-solid (VSS) depending on the physical state of the metal droplet, which is often gold. In such mechanism, monolayers (MLs, may include two sublayers of elements belonging to a single column of the periodic table, e.g., Zn and S, or Ga and As) are stacked along the [111]—cubic or [1]—hexagonal direction after crystallization at the catalyst-nanowire interface.

In the past, ZnS nanostructures have been synthesized using various techniques, mainly by vapor phase transport from high purity ZnS powder [19,20], but also chemical vapor deposition (CVD) [21], laser ablation [22], and metalorganic chemical vapor deposition (MOCVD) [23]. Growth in solution has been also investigated, and recently, Liu et al. have reported on solution–solid–solid ZnS NWs prepared at low temperature in the range of 120–200 °C [24]. Many of the experiments have resulted in mixed and randomly aligned 1D nanowires and nanobelts. In order to achieve uniform and vertical arrays of nanowires, it appears crucial to accurately select the nature and the orientation of the substrate. GaAs (111)B oriented wafers were found to be very suitable for the MOCVD growth of vertical Ga-assisted ZnS NWs with length around 3–4 µm and diameter in the range of 80–200 nm [23]. On the other hand, horizontal ZnS NWs have been produced following a surface-guided growth on sapphire substrates with different orientations, namely C (0001), M (1-100), and R (1-102) [25].

The structural properties and crystal phases of ZnS nanostructures have been investigated by transmission electron microscopy (TEM). Like III-Vs [26,27] and other II-VI semiconductors [28], ZnS nanowires are most often grown along [111] direction with ZB structure, or along [1] with WZ structure, and twins and stacking faults are commonly observed perpendicular to the growth direction [29,30]. However, regarding nanoribbons and nanobelts, i.e., flat nano-objects, they show WZ structure along [01-10] axis [31], and possibly with ZB inclusions [20]. In nanobelts, it has been observed that the interface between cubic and hexagonal segments (i.e., the stacking fault plane) is always parallel to the growth direction [19].

In nanowires, twins can emerge periodically, as observed by Hao et al. [20] in a ZB ZnS structure with a frequency every seven to nine Zn-S layers. Jiang et al. [22] also described ordered stacking faults resulting in a polytype modulated nanostructure composed of 8H and 10H half-cells. Very recently, we reported on the identification of 15R polytype in VLS ZnS NWs grown on GaAs (111)B [17].

In this work, we investigated the catalyzed growth of ZnS NWs on GaAs (100), (111)A, and (111)B substrates, as well as on ZnS (buffer) pseudo-substrates, by using MOCVD. Beyond simply listing a variety of nanostructures, we aimed to give an original contribution and discussion on NWs growth mechanisms, control of crystal phase, and, more generally, on the understanding of polytypism in semiconductor nanowires. We first carried out basic studies on ZnS thin films to better know about MOCVD growth regimes and kinetics. Subsequently, we checked the formation of Au droplets on the different substrates, with and without ZnS buffer. Then, we carefully investigated the growth parameters leading to the best quality of ZnS NWs in terms of vertical alignment, uniformity, and crystal phase. TEM experiments allowed us to unambiguously identify ZnS polytypic phases in nanowires: 3C, 2H, and 15R. Herein, the structural results are analyzed on the light of the literature in the field, and correlated with the growth conditions: substrate temperature, VI/II ratio, supersaturation, and physical state and composition of the droplet. We especially highlight twinning superlattices in zinc blende ZnS NWs. It is emphasized that our experimental results agree with theoretical work on III-V NWs growth.

## 2. Materials and Methods

ZnS thin films and nanowires were grown by MOCVD in a vertical reactor operating at atmospheric pressure. In our system, the reactor walls are water cooled at 20 °C to lower parasitic reactions. The substrate holder, made of SiC-coated graphite, is heated by electromagnetic induction. The holder is inclined so that the sample surface makes an angle of 45° with respect to the gas flow direction.

High purity diethylzinc (DEZn) and ditertiarybutylsulfide (DTBS), stored in stainless steel bubblers and maintained at 17 °C, were used as the zinc and sulfur metalorganic (*MO*) precursors, respectively. Hydrogen was the carrier gas passing through the bubblers and allowing the dilution of the *MO* flows. In the reactor, the *MO* partial pressure above the substrate is controlled by the simple equation:

$$P_{MO}=\frac{P_{vap}\left(MO\right)\cdot Flux\left(MO\right)}{Total\;flux}$$

where *P_vap_ (MO)* is the vapor pressure above the liquid *MO*, and *Flux (MO)* is the H_2_ flux entering the bubbler. The total flux in the reactor adds *MOs* as well as pure H_2_ dilution flows.

For basic growth studies on ZnS thin films, we used standard GaAs (100) substrates. At first, after introduction in the reactor, substrates were deoxidized at 550 °C for 10 min under a flow of H_2_. This is a typical process used in MOCVD. Deoxidation at a temperature higher than 550 °C damaged the GaAs substrate. Two series of ZnS layers were made: (i) varying growth temperatures in the range 320–570 °C while maintaining constant DEZn and DTBS partial pressures at a fixed ratio R_VI/II_ = 2, and (ii) varying VI/II ratio between 0.6 and 3.2 keeping growth temperature at 450 °C. All of these samples underwent a 45-min growing period.

On another hand, ZnS nanowires were grown by introducing substrates coated with a gold layer. In that case, GaAs (100), (111)A, and (111)B substrates, as well as ZnS (buffer)/GaAs pseudo-substrates were used. For one experiment, GaP (111) was also tested. The thin Au films were deposited on the substrates using an Edwards Auto306 thermal evaporator. The Au thickness was typically around 1 nm. The dewetting process leading to the formation of Au-droplets on the substrate surface was thus done in situ under hydrogen flow, typically at 500 °C for 10 min. A list of all the grown samples, thin film and nanowires, is given in Table 1 with their growth conditions.

After growth, the nanostructures were observed using scanning electron microscopy (SEM, JEOL 7001F). Transmission electron microscopy (TEM) enabled to determine the crystalline structure of the NWs, after scratching and spreading them on a TEM copper grid covered with a holey carbon film. The grid was inserted in a Zeiss Libra 200 MC TEM/STEM equipped with a monochromator and an in-column filter. The microscope was operated at 200 kV to achieve a lattice resolution below 1.5 Å in high resolution mode. X-ray diffraction analysis was performed using a Seifert diffractometer.

## 3. ZnS Thin Films: MOCVD Basic Studies

In the past, we studied the thermal decomposition of the diethyl–Zn organometallic molecule, by using a quadrupole mass spectrometer coupled with an isothermal reactor. Experimental details and results are reported in reference [32]. Under hydrogen atmosphere, DEZn starts to decompose at 300 °C, following a two-step homolytic fission.

(C_2_H_5_)_2_Zn => C_2_H_5_*Zn + C_2_H_5_*
C_2_H_5_*Zn => Zn + C_2_H_5_*
2C_2_H_5_* => C_4_H_10_ or 2C_2_H_5_* => C_2_H_6_ + C_2_H_4_ (1)

The complete decomposition is achieved at 360 °C. The activation energy is calculated around 51 kcal/mol. The recombination of ethyl radicals gives the hydrocarbons by-products, ethylene and ethane, which are desorbed from the surface and sent to the reactor exhaust. In the same way, the thermal decomposition of ditertiarybutyl–sulfide (C_4_H_9_-S-C_4_H_9_) had been preliminary investigated (unpublished work). We found that DTBS alone decomposes between 500 °C (onset) and 550 °C (end). In the presence of a Zn–organometallic, due to the co-pyrolysis effect [33], the decomposition was shifted toward low temperatures, between 350 and 400 °C.

Hence, from the decomposition kinetics of the organometallics, we expect the optimum temperature conditions for the MOCVD growth of ZnS to be in the range of 400–500 °C.

### 3.1. MOCVD Growth Regimes

**Figure 1:**
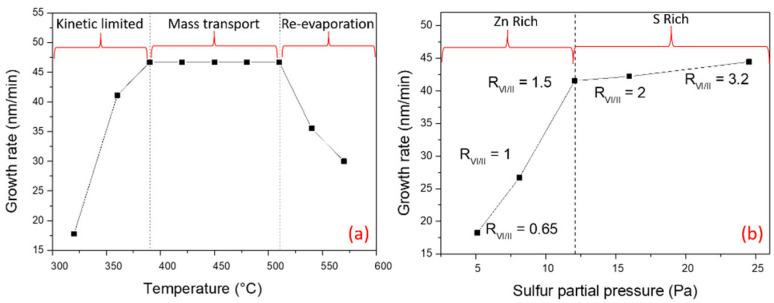
Growth rate of ZnS thin films as a function of: (**a**) substrate temperature, and (**b**) DTBS partial pressure.

A first series of samples was made at various growth temperatures between 320 to 570 °C (samples TF1-TF9, see Table 1). In Figure 1a, the growth rate is plotted as a function of the substrate temperature, and we can identify three distinct growth regimes for MOCVD [34]: (a) a kinetically limited regime between 320 °C and 390 °C, governed by surface reactions; (b) a mass transport regime in the range of 400–510 °C, where species diffuse through the boundary layer just above the substrate, and (c) a re-evaporation regime, where adatoms desorb from the surface. The mass transport growth is characterized by high growth rates and a weak dependence on the substrate temperature. For semiconductor thin films, it is likely that the majority of MOCVD processes will take place in such a regime, where it is easier to control the growth rate. In the case of ZnS, 400–500 °C appears as a suitable range, in agreement with the decomposition studies of DEZn and DTBS.

The second series of ZnS thin films was fabricated to identify the MOCVD regimes for various precursor fluxes, similar to the temperature investigation. The sulfur partial pressure was changed between 5.1 and 25 Pa while the zinc partial pressure and temperature remained constant at 7.8 Pa and 450 °C, respectively. Figure 1b depicts two growth regimes that emphasize the influence of sulfur precursor on the growth rate. In a first regime (low sulfur flows in the range between 5.1 and 11.7 Pa), the growth rate increases linearly with the sulfur flow, meaning that the growth proceeds under zinc-rich conditions. The growth rate starts to saturate when the sulfur flux supply rises over 11.7 Pa (R_VI/II_ = 1.5), and this is referred to as a sulfur-rich regime where now the zinc supply regulates the growth rate. Interestingly, it is noteworthy that the transition between Zn-rich and S-rich regimes does not correspond to a precursor ratio of 1. This indeed may depend on the decomposition rate of each organometallic source at a given temperature. For the subsequent development of ZnS NWs (1D growth), it is important to have identified such sulfur- and zinc-rich regimes.

In conclusion, basic studies on ZnS thin films growth enabled to identify mass-transport regime and zinc- (sulfur-) rich conditions.

### 3.2. X-ray Diffraction Analysis of ZnS Buffer Layers

**Figure 2:**
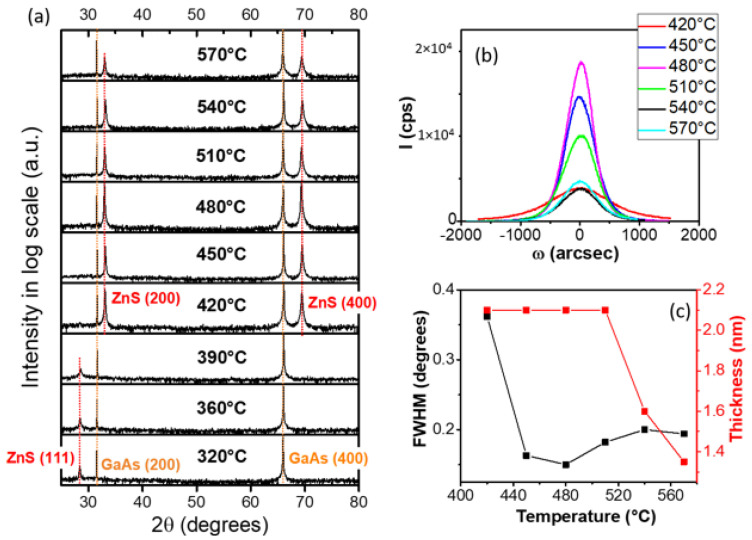
X-ray diffraction analysis of ZnS layers grown at various temperatures, from 320 °C to 570 °C: (**a**) θ-2θ scans, (**b**) ω-2θ rocking curves, and (**c**) full width at half-maximum and layer thickness plots, as a function of the growth temperature.

The crystalline properties of ZnS thin films were studied, with the aim to assess and optimize the quality of the ZnS buffer layer on which the gold catalyst droplet will lay, and hence will trigger the NW growth. Figure 2a gathers XRD measurements (θ-2θ scans) of ZnS layers grown at different temperatures between 320 and 570 °C. In all spectra are seen the GaAs substrate diffraction peaks, namely (002) and (004), at 2θ = 31.6° and 66°. For the lowest growth temperatures (from 320 to 390 °C), a rather broad peak observed at 2θ = 28.4° is attributed to ZnS (111) plane diffraction. It is clear that increasing the growth temperature above 400 °C changes the film orientation, which turns to (001) plane. The intensity of the (002) diffraction at 2θ = 33° increases with increasing the temperature up to 480 °C. In Figure 2b, the variation of the full width at half maximum (FWHM) of ZnS (002) diffraction peak is plotted as a function of the growth temperature. We find a minimum FWHM around 480 °C. Thus, XRD measurements indicate an improvement in crystallization up to 500 °C. Above 510 °C, due to re-evaporation of the adsorbed species, the thickness strongly decreases and the comparison of crystal quality is not relevant.

In the same way, we performed XRD measurements (not shown here) of the ZnS thin films grown at different sulfur partial pressure, between 5.1 and 25 Pa, at Tg = 450 °C, and keeping constant P_DEZn_ = 7.8 Pa. This enabled a variation of R_VI/II_ in the range of 0.6–3.2. The analysis led to an optimized VI/II ratio of around 2, i.e., in a sulfur-rich regime. Therefore, for the deposition of the ZnS buffer on GaAs and GaP substrates, we will set the growth temperature at 450 °C (in the mass-transport regime), and R_VI/II_ = 2 (corresponding to sulfur-rich conditions).

## 4. ZnS Nanowires Catalyzed Growth

After having studied MOCVD growth conditions for ZnS thin films, we now come to Au-catalyzed ZnS 1D nanostructures. In the following section, we will carefully investigate the droplet formation, and the growth parameters leading to arrays of ZnS NWs with the best verticality and uniformity.

### 4.1. Au-Droplet Formation

The VLS and VSS processes involve catalyst droplets on the substrate or pseudo-substrate surface. In the following section, we investigate the formation of gold nanoparticles on GaAs (100), GaAs (111)B, and ZnS/GaAs (111)B.

**Figure 3:**
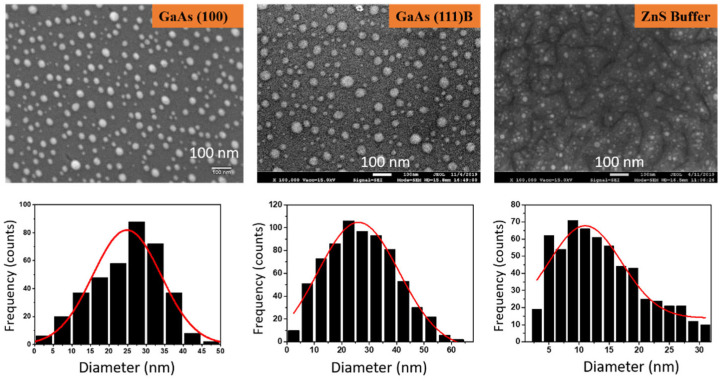
SEM images and statistics of gold droplets deposited and dewetted on GaAs (100), GaAs (111)B, and ZnS/GaAs (111)B substrates.

A thin Au layer of 1 nm is first deposited on the substrates. Subsequently, a dewetting process (annealing at high temperature) initiates the formation of nanoparticles favored by Ostwald ripening and island coalescence [35]. The dependence of nanoparticle size and distribution on the film thickness, annealing duration, and annealing temperature has been well studied in the past on different substrates [36,37]. The size of gold nanoparticles is directly proportional to the thickness, annealing duration, and temperature, whereas the density is inversely proportional. However, the statistics of Au droplet on ZnS buffer are still to be drawn. Herein, the size distribution and the density of Au nanoparticles on different substrates were assessed after the analysis of SEM images and using ImageJ software as illustrated in the Figure 3. For GaAs (100), the nanoparticle diameter expands from 3 to 48 nm with an average of 25 nm and a density of 350 particles/µm^2^ is calculated. On GaAs (111)B, the density is lower, 240 particles/µm^2^, and the range of nanoparticle size is broader, between 3 and 62 nm, but the average remains around 25 nm. On ZnS buffer layer, the dewetting process leads to quite different statistics, as the size distribution is measured between 3–32 nm (average 13 nm, i.e., half of the value on GaAs surface) with a higher density of 600 particles/µm^2^. Consequently, the mean size of nanoparticles on bare GaAs is larger compared to the one on ZnS surface. This is due to the interaction between the gold film and the substrate. Such interaction leads to the interdiffusion of Ga atoms from the substrate to the Au nanoparticles and produces Au-Ga alloyed nanoparticles [38]. The phase of the alloyed catalyst is dependent on the composition of Ga present in the catalyst. Different metallic compounds have been identified and reported in the past, such as hexagonal β′Au_7_Ga_2_, and orthorhombic AuGa or Au_2_Ga [38,39].

### 4.2. Growth Parameter Studies

**Figure 4:**
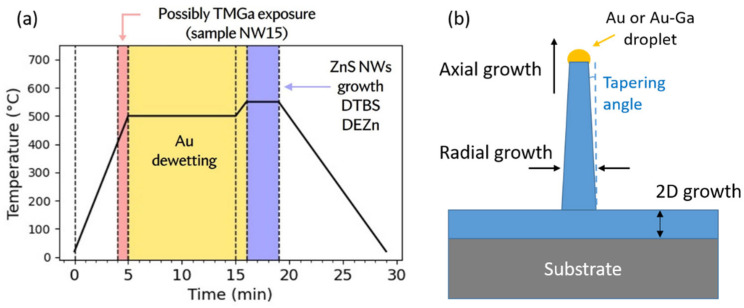
(**a**) Typical growth diagram, note that the TMGa flux is only applied for sample NW15, and (**b**) Schematic of NW growth, distinguishing axial, radial and 2D growths.

Substrate temperature, flux ratio, supersaturation, or buffer layer are the key parameters as regards the growth of III-Vs and II-VIs NWs [40,41]. Figure 4a gives the typical growth diagram, and Figure 4b illustrates the emergence of a catalyzed nanowire, as the result of the competition between 2D growth on the substrate, 1D (axial) growth below the droplet (pure Au or Au-Ga alloy), and radial growth on the sides of the NW. NWs were grown after the gold dewetting process. In addition, a flow of trimethylgallium (TMGa) was possibly supplied for a short period of 1 min at the end of the temperature ramp (sample NW15, the use and role of TMGa will be explained in Section 5.3). As mentioned in the state of the art, there are not so many papers dealing with Au-assisted ZnS NWs grown by MOCVD. Hereafter, we take this opportunity to carefully investigate the role of growth parameters.

**Figure 5:**
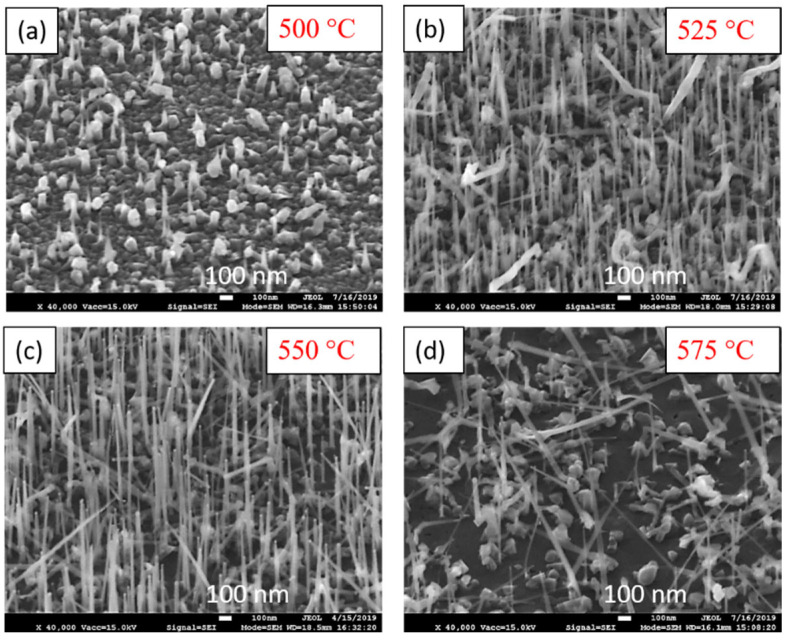
45°-tilted SEM image of samples prepared on GaAs (111)B sustaining R_VI/II_ = 2 at different temperatures: (**a**) 500 °C, (**b**) 525 °C, (**c**) 550 °C and (**d**) 575 °C.

The first step was to study the effect of temperature on the growth of ZnS NWs and a series of experiments was performed increasing the substrate temperature from 450 to 575 °C. The growth duration was 3 min for all samples (S flow: 15.6 Pa, Zn flow: 7.8 Pa and ratio R_VI/II_ = 2, samples NW1 to NW5, see Table 1). At the lowest temperature, 450 °C, the growth produced a rough ZnS layer which entirely covered the catalyst, and hence no NWs were observed (not shown here). Figure 5 gathers 45°-tilted SEM images of samples prepared on GaAs (111)B at different temperatures: (a) 500 °C, (b) 525 °C, (c) 550 °C and (d) 575 °C. It appears that the growth of NWs just initiates at 500 °C but still forming a rough layer with 3D objects on the surface (Figure 5a). The increase of the temperature at 525 °C and 550 °C, Figure 5b,c, reinforces the growth of vertical and more homogeneous NWs. The maximum length is around 2 µm, and diameter is in the range of 10–50 nm, even if it is challenging to accurately measure from SEM images. These latter give an idea of the presence and vertical alignment of the NWs, and TEM experiments will provide more information on droplet and NW diameters in the next section. Some NWs are nevertheless inclined, and 3D objects are still present on the surface. Further increase in the temperature, at 575 °C, yields rather long but disordered NWs and the density is lower (Figure 5d).

Interestingly, if one refers to the Figure 1 presenting the three growth regimes for ZnS films, the emergence of ZnS NWs occurs in the re-evaporation regime. In the mass-transport regime, the droplet is buried in the growing layer. This could be due to equivalent axial and 2D growth rates (illustrated in Figure 4b). In the re-evaporation regime, zinc and/or sulfur atoms desorb from the substrate surface, decreasing strongly the 2D rate. Then we can assume that, on the contrary, the diffusion of species in the catalyst-droplet is enhanced by the temperature, and this is why the axial growth rate increases. In a previous work on ZnO NWs growth, we have demonstrated the effect of the gold droplets, which efficiently catalyze the adsorption of the zinc precursor (DEZn) to initiate the growth of nanostructures [42]. Here, we expect the same behavior for ZnS NWs; i.e., a preferential adsorption and diffusion of the precursor species at the catalyst particle (Au or Au-Ga alloy) instead of their re-evaporation. Therefore, in such temperature conditions, the axial growth rate below the catalyst droplet is significantly higher than the 2D growth rate on the substrate, hence fostering the NW morphology (as shown in Figure 4b).

**Figure 6:**
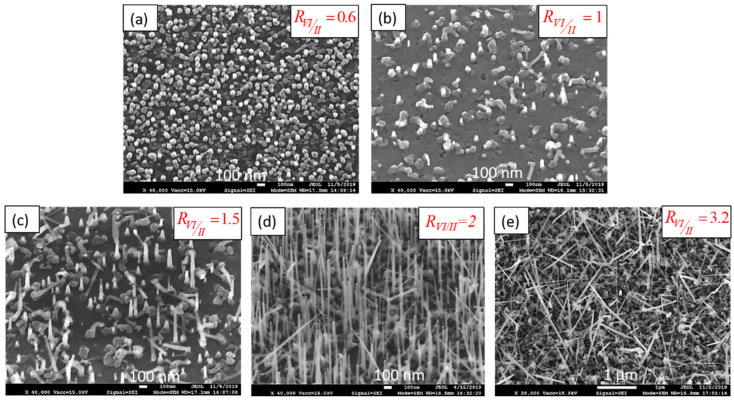
45° tilted SEM images of ZnS NWs with various VI-II ratio (**a**) 0.6, (**b**) 1, (**c**) 1.5, (**d**) 2, and (**e**) 3.2.

We continued with the study of VI/II ratio (R_VI/II_). Another series of samples was prepared varying the precursor partial pressure ratio at 550 °C, as this temperature was considered as the optimized one (see Table 1, samples NW6 to NW9). The SEM images of the NWs grown on GaAs (111)B varying R_VI/II_ are shown in Figure 6. Taking into account the preliminary study of ZnS layers in Section 3.1, we remind that VI/II ratios of 0.6 and 1 correspond to zinc-rich conditions, 1.5 is the equilibrium point, and 2 and 3.2 correspond to sulfur-rich growth. It is clear from the SEM images that zinc-rich regime (R_VI/II_ = 0.6 and 1, Figure 6a,b) does not enable the growth of NWs, but rather leads to dots and 3D objects on the surface. An increase of the sulfur partial pressure initiates the 1D growth (R_VI/II_ = 1.5 in Figure 6c), as short NWs are observed, with lengths up to a few hundred nanometers. Setting R_VI/II_ = 2 (Figure 6d) produces the best ZnS NWs. They are 1–2 µm long, and most are vertically aligned. Further increase in the sulfur flow (R_VI/II_ = 3.2, Figure 6e) leads to longer NWs, above 2 µm, but appears to disturb the vertical orientation. These results show that zinc-rich conditions (refer to Figure 1) suppress the growth of NWs whereas sulfur-rich conditions yield long and vertical NWs, with an optimized VI/II ratio around 2. A similar influence of precursor ratios was observed for ZnTe NWs [41], and ZnSe NWS [43] grown by molecular beam epitaxy, i.e., an excess of group VI element is required. We have no explanation for that, especially since another group found the opposite behavior in ZnSe NWs [44], i.e., worm-like and kinked in selenium-rich conditions, but straight in zinc-rich.

**Figure 7:**
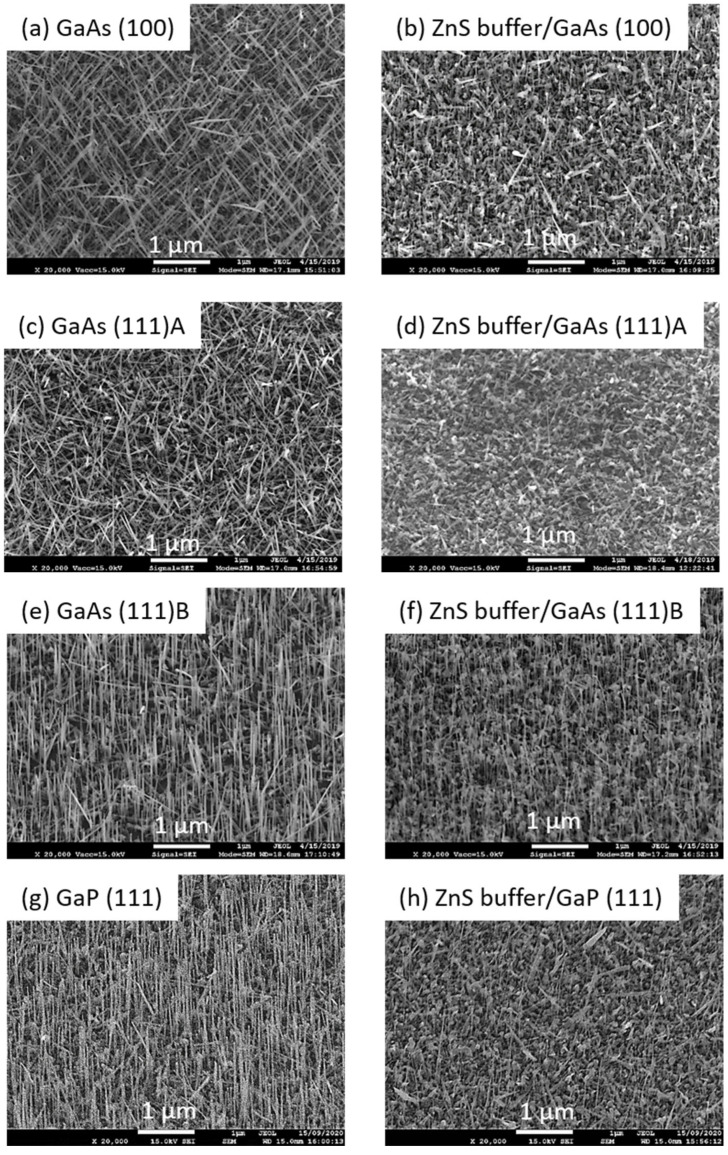
45° tilted SEM images of ZnS NWs grown on: (**a**) GaAs (100), (**b**) ZnS/GaAs (100), (**c**) GaAs (111)A, (**d**) ZnS/GaAs (111)A, (**e**) GaAs (111)B, (**f**) ZnS/GaAs (111)B, (**g**) GaP (111), and (**h**) ZnS/GaP (111).

After optimization, NWs growth was also performed on a set of different surfaces, namely GaAs (100), GaAs (111)A, GaAs (111)B, GaP (111), and ZnS buffers deposited on such substrates. SEM images are gathered in Figure 7. The growth on GaAs (100) and GaAs (111)A substrates resulted in randomly oriented ZnS NWs (Figure 7a,c). The best uniform and vertical arrays of NWs were produced on GaAs (111)B surfaces, as shown in Figure 7e, in agreement with experimental works reported in the literature. Introducing ZnS buffer leads to a mitigated effect concerning GaAs (100) and GaAs (111)A substrates, with no strong improvement in the quality of the NWs arrays as they still appear inclined (Figure 7b,d). On ZnS(buffer)/GaAs (111)B pseudo-substrate, nanowires grow vertically like in the case of bare GaAs (111)B, but noticeably, they are much thinner, and therefore are more difficult to observe using SEM (Figure 7f). A few 1D nanostructures appear much broader at the base, and are possibly flat, like very elongated triangles. In this experimental work, GaP (111) surfaces were also tested. As ZnS is nearly lattice matched to GaP, it was interesting to study whether arrays of ZnS grown on ZnS (buffer)/GaP would show a strong improvement regarding uniformity and verticality. SEM images are given in Figure 7g (bare GaP) and Figure 7h (with ZnS buffer). Actually, the results on GaP (111) were found very similar to the case of GaAs (111)B, including TEM results and structural analysis of single NWs. Therefore, in the following sections, we will focus on NWs grown on GaAs (111)B and on ZnS (buffer)/GaAs (111)B.

### 4.3. Au-Droplet Size and Nanowire Diameter

**Figure 8:**
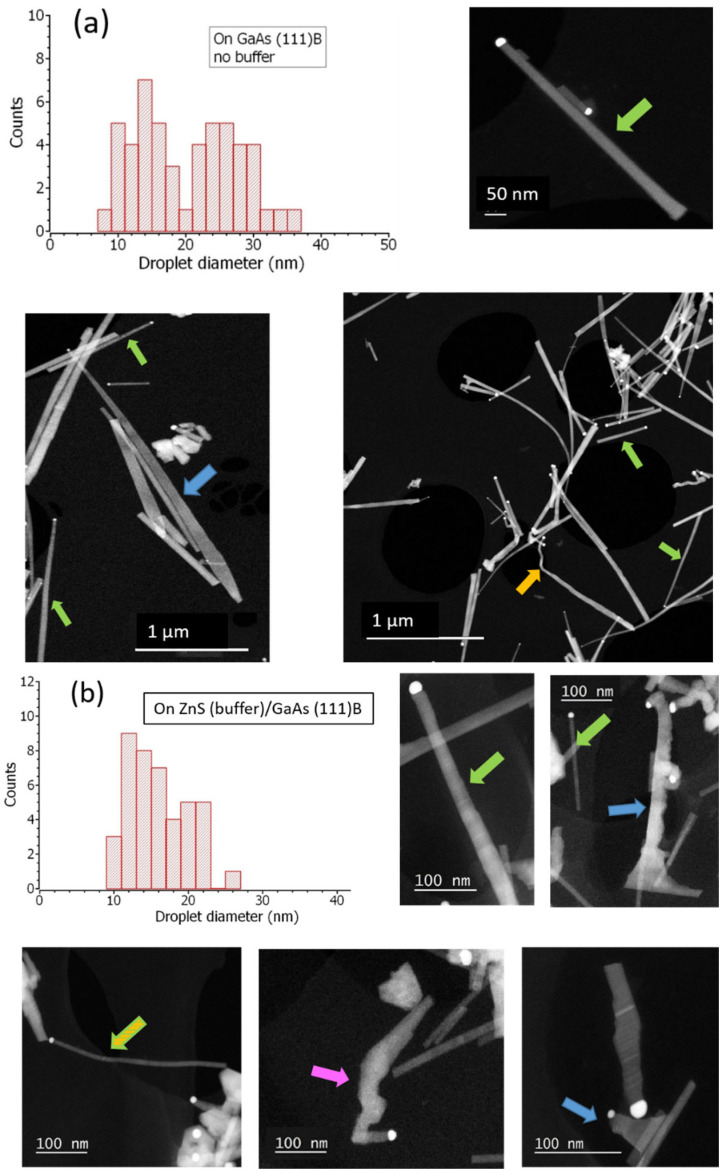
STEM/HAADF image and droplet diameter statistics of ZnS NWs grown on: (**a**) GaAs (111)B, and (**b**) ZnS (buffer)/GaAs (111)B.

To get a better idea of the NWs morphology, Figure 8 gathers high angle annular dark field (HAADF) scanning TEM (STEM) images taken on two representative samples grown on GaAs (111)B and ZnS (buffer)/GaAs (111)B. Different kinds of 1D nanostructures can be distinguished, with a bright catalyst droplet on their top. The most important population concerns long and straight nanowires, as indicated by the green arrows. At the tip, their diameter is more or less equal to the droplet, but it weakly increases going toward the base of the nanowires, making them slightly tapered. The tapering angle is small, around 0.5°. Another family of 1D nanostructures presents a much wider base, and the tapering is more pronounced (1.2° in the case of the NW indicated by a blue arrow in Figure 8a). Actually, such NWs could be flat and hence be nanoribbons [31]. Other ZnS nano-objects appear as worm-like structures (pink arrow), or kinked nanowires (orange arrow).

In addition, we seized the opportunity to present statistics on the droplet diameter observed at the tip using TEM images. Two statistics are given in the Figure 8, corresponding to a collection of ~50 NWs whether grown on GaAs (111)B or ZnS (buffer)/GaAs (111)B. On bare GaAs (no buffer), the droplet diameter spans from 7 to 36 nm. The histogram actually shows two populations, one in the range of 7–20 nm (average~15 nm), and the other in the range of 20–36 nm (average~28 nm). On ZnS (buffer)/GaAs (111)B, the droplet diameter varies between 9 and 26 nm. Here the tendency of forming two populations is not as clear, and the mean size is ~16 nm. In both cases, the histograms are quite different from the ones presented in Figure 3, before the NW growth (i.e., just after dewetting). Indeed, the parts corresponding to the largest droplets are absent in the new histograms, letting us infer that the small nanowires were preferred during the growth of ZnS NWs.

## 5. Structural Characterization by TEM and Discussion of the Growth Mechanisms

In this section we investigate by TEM the structural properties of the grown ZnS nanowires, focusing on the long and straight population.

### 5.1. VLS versus VSS

In a recent work, we compared the structural properties and crystal phases of ZnS NWs whether grown on bare GaAs (111)B substrate, or ZnS (buffer)/GaAs (111)B pseudosubstrate [17]. To do so, we used transmission electron microscopy, and details are given in this article. On bare GaAs (111)B, due to the diffusion of Ga into Au particles during dewetting, the catalyst droplets are expected to be in the liquid state during the growth of ZnS NWs (at 500–550 °C), so that the mechanism will follow the vapor-liquid-solid mode. On the other hand, the use of ZnS buffer inhibits the diffusion of Ga from the substrate and pure Au-droplets are formed after dewetting. In that case, the growth mechanism will be vapor-solid-solid, as it will involve a solid droplet (gold melting point at 1064 °C).

**Figure 9:**
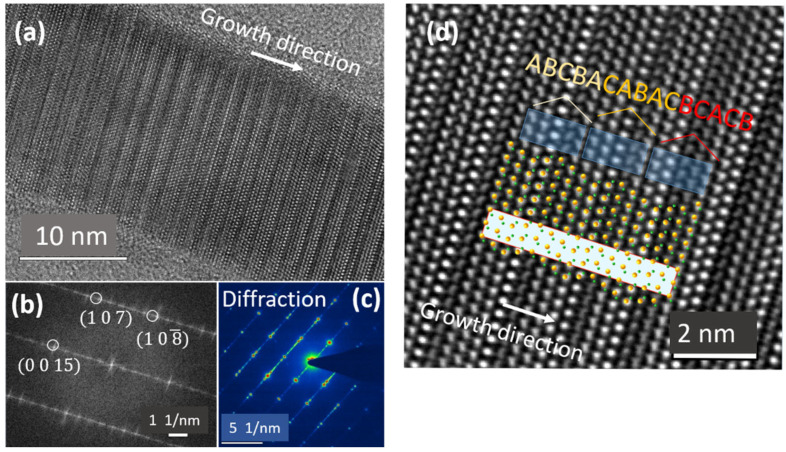
(**a**) HRTEM image of the nanowire, (**b**) the related FFT pattern, (**c**) SAED pattern from a single NW (**d**) Fourier-filtered image superposed with the 15R structure model and the respective stacking sequence ABCBA–CABAC–BCACB. (**a**,**b**,**d**) are reprinted with permission from Ref. [17]. Copyright {2021}, Springer Nature.

Figure 9 illustrates the TEM analysis of VLS ZnS NWs (i.e., without buffer). Remarkably, we have identified large 15R segments of ZnS. Fast-Fourier transform (FFT) pattern and selective area electron diffraction (SAED) pattern attest for the 15R crystal structure (Figure 9b,c). In this polytype, the repeated sequence corresponds to ABCBA-CABAC-BCACB planes, and it can be confirmed by superimposing a 15R model on the Fourier-filtered image of nanowire as shown in Figure 9d.

**Figure 10:**
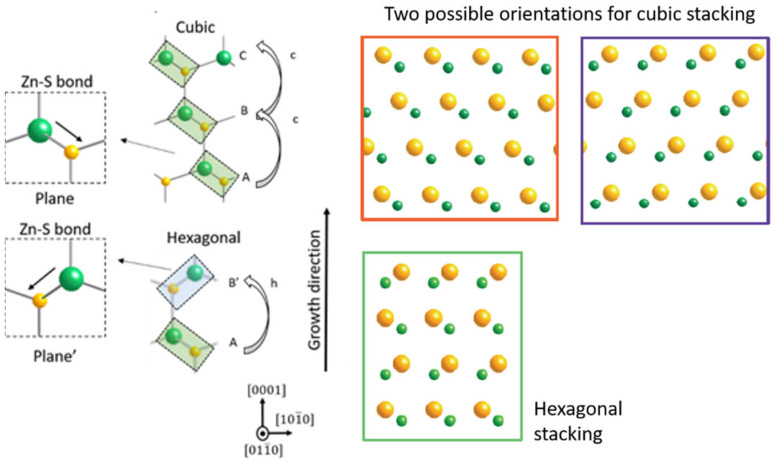
Notation of the planes with respect to the direction of the Zn-S bond not parallel to the growth direction: the stacking gives either hexagonal *h* or cubic *c* planes.

Let us explain the formation of polytypes in ZnS NWs, and more generally, in semiconductor NWs. Along the [111] or [1] direction, a ML can be seen as a cubic or a hexagonal plane, in our case depending on the orientation of the Zn-S bonds not normal to the growing plane. This is illustrated in Figure 10. A newly deposited layer will be called ‘*c*’ if such Zn-S bonds are parallel to the corresponding ones of the previously stacked layer. It will be called ‘*h*’ if not parallel. In ZnS, the very low experimental stacking fault energy, less than 6 mJ/m^2^ (45 mJ/m^2^ in GaAs) [45], allows for an easy switch from forming a new hexagonal *h* layer or forming a new cubic *c* layer. Actually, this just corresponds to an in-plane rotation of 60°. Stacking *c* planes leads to the ABCABC cubic structure, while stacking *h* planes generates ABAB hexagonal phase. When periodic sequences of *c* and *h* layers are deposited, this gives rise to high order polytypes. As an example, repeated sequence *chch* leads to the 4H crystal phase. For 15R, the repeated stacking sequence is *hcchc–hcchc–hcchc*.

Regarding VSS ZnS NWs (i.e., with buffer), the situation is quite different. A TEM analysis on VSS ZnS NWs fabricated in the same MOCVD run (with VLS ZnS NWs analyzed in Figure 9) but grown via solid Au droplets can be found in Ref. [17]. Pure WZ and ZB domains are revealed, and they frequently switch between these two structures.

### 5.2. Controlling Crystal Phases: The Key Role of Supersaturation

In semiconductor NWs, random stacking faults and uncontrolled polytypism are commonly observed. These defects have an adverse effect on the optical and electrical properties, lowering the quantum efficiency, carrier lifespan, and mobility of carriers. Therefore, it is crucial to explore all growth conditions to achieve and control the best crystal quality.

In this section, we pursue the idea that supersaturation in the catalyst droplet plays a major role and gives an explanation behind the formation of polytypes in SC nanowires: the WZ phase occurs at high supersaturation while ZB occurs at the beginning of the growth when supersaturation stays low [46,47].

Herein the MOCVD process, we controlled the supersaturation through the variation of Zn and S fluxes. We could reduce Zn and S partial pressures by four times compared to the reference samples and hence allowed NWs to grow in minimum supersaturation conditions. Below these partial pressures, no homogenous deposition was observed on the samples. On the other hand, we could increase the precursor fluxes by 1.5 times (reaching the maximum flow allowed by the DTBS mass-flow controller) with the aim of imposing higher supersaturation. We thus assume that varying precursor partial pressures lead to a modulation of the supersaturation in the catalyst particle. This assumption seems relevant in the case of a liquid droplet, where elements would diffuse.

**Figure 11:**
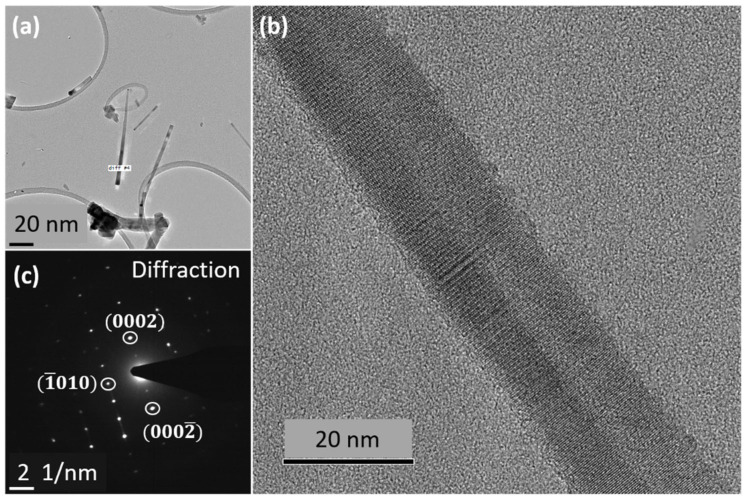
(**a**) bright-field TEM image of ZnS NWs (high supersaturation), (**b**) the HRTEM image of NW revealing a pure WZ phase, (**c**) SAED pattern of the NW presenting WZ-like footprint.

We performed TEM experiments on several NWs grown with higher supersaturation (see conditions of sample NW13 in Table 1). The bright-field (BF) TEM image of a single NW is illustrated in Figure 11a. The NW has a conical tapered shape. The diameter is 10 nm just below the droplet and 25 nm at the bottom, suggesting a significant lateral growth rate. In Figure 11b, we present the high resolution (HR) TEM image of the NW. Remarkably, the NW is defect-free and exhibits a pure WZ structure. The SAED pattern in Figure 11c confirms the pure WZ phase. Hence, an increase in the Zn and S fluxes has changed the crystal phase from 15R to WZ.

**Figure 12:**
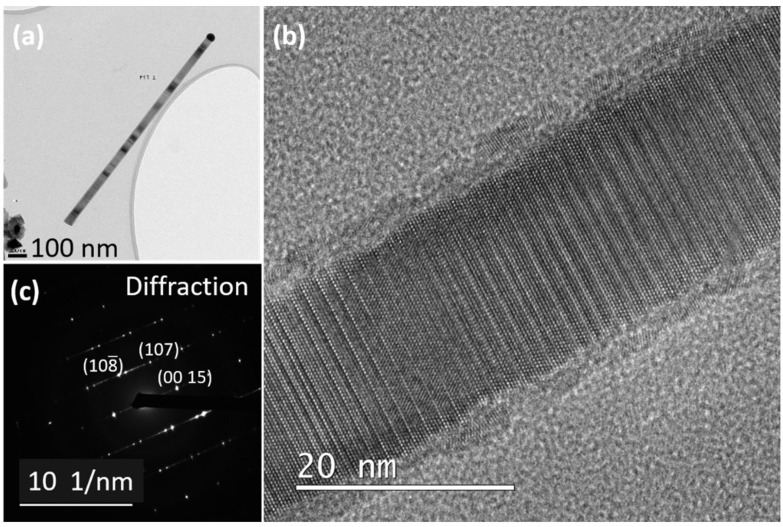
(**a**) Bright-field TEM image of a single NW grown with lower supersaturation, (**b**) HRTEM image of NW revealing a 15R phase, and (**c**) SAED pattern of the NW shows a 15R footprint.

In the next experiment, ZnS NWs were grown at a lower growth rate (sample NW14) by decreasing the fluxes of Zn and S precursors, four times less than the reference sample. The structural properties and crystal phase were again studied using bright field, diffraction, and HRTEM. The bright-field TEM image of a single NW taken in <010> zone axis is shown in Figure 12a. The catalyst droplet can be clearly seen at the top with a darker spherical contrast. The NW diameter just below the catalyst is 45 nm and 50 nm at the bottom making it almost a perfect cylindrical NW. This small difference in diameter implies a decrease in the lateral growth using a low growth rate, compared to the cases of both reference and high Zn-S fluxes samples.

In Figure 12b, the HRTEM image provides visuals of a 15R crystal phase stacking. The diffraction image in Figure 12c confirms the typical diffraction pattern of a 15R structure observed before for the reference sample. Additionally, the 15R would have an improved tendency at low growth rate, as it appears to dominate with less stacking faults.

**Figure 13:**
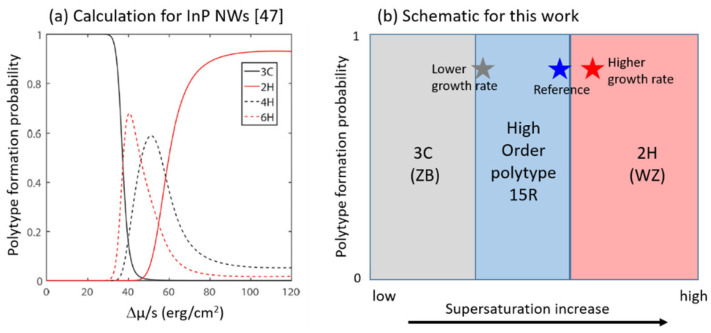
(**a**) Polytype formation probability as a function a supersaturation in InP NWs, reprinted with permission from Ref. [47]. Copyright {2016}, American Chemical Society, and (**b**) Illustration of supersaturation effect on polytype formation probability in ZnS NWs. The gray, blue, and red stars correspond to ZnS NWs experiments with various growth rates.

Let us now discuss these results in the light of previously reported theoretical works. In particular, Johansson et al. [47] calculated and plotted the formation probabilities of GaAs and InP polytypes as a function of supersaturation (Δµ) in the catalyst droplet, in the framework of classical nucleation theory and anisotropic next nearest neighbor Ising (ANNNI) model. In the graph reproduced in Figure 13a for InP NWs, three domains are defined. At low Δµ, the cubic phase 3C is predicted, with 100% probability. At high Δµ, the hexagonal structure 2H is expected with the highest probability. In between, and depending on the interaction between the forming nucleus and the previously grown layers (namely the *J_i_* parameters of the ANNNI model), a window of supersaturation opens for the occurrence of high order polytypes (HOP) such as 4H and 6H. Even though the theoretical work was done for III-V compounds, we can expect the same tendency for ZnS. Hence, in Figure 13b, an illustration of the supersaturation effect on polytype formation probability is proposed, where the grey, blue, and red stars correspond to our ZnS NWs experiments with various growth rates. Interestingly, our TEM observations above show that we turned the crystal phase of ZnS NWs from 15R to 2H by increasing supersaturation, thus in full agreement with these theoretical predictions. This is illustrated by the blue and red stars. We notice that a relatively small increase in the growth rate (by 1.5) has brought a pure WZ phase, so that the reference 15R sample may have been at the upper limit of the HOP window. In the case of our ZnS NWs grown with lower supersaturation, we were expecting a possible ZB crystal phase, which would indeed follow the predicted tendency. This is not the case, as the 15R phase is still found (see the grey star in Figure 13b). One explanation could be that the HOP window is much larger for ZnS and/or the 3C domain is much reduced for ZnS as compared to InP or GaAs. This can be understood by the large difference in stacking fault energy between these materials, as mentioned in Section 5.1, so that in ZnS the occurrence of high order polytypes and 2H crystal phase is favored at the expense of the cubic structure.

### 5.3. Twinning Superlattice Highlight in ZnS NWs

In Section 5.1, we underlined how the state and composition of the catalyst particle may change the growth mechanism and consequently the crystal phase of the growing NW. Indeed, theoretical models have demonstrated how the surface energy and contact angle of the droplet impact the nucleation of hexagonal or cubic planes, leading to a possible control of the phase, between ZB and WZ [48]. Herein, we aimed to increase the Ga content in the catalyst. Actually, this can be easily done and controlled by exposing the Au-particles to a trimethylgallium (TMGa) flux after dewetting. Hence, in the MOCVD reactor, it is possible to intentionally “feed” the droplet with Ga atoms, before growing the ZnS NWs.

**Figure 14:**
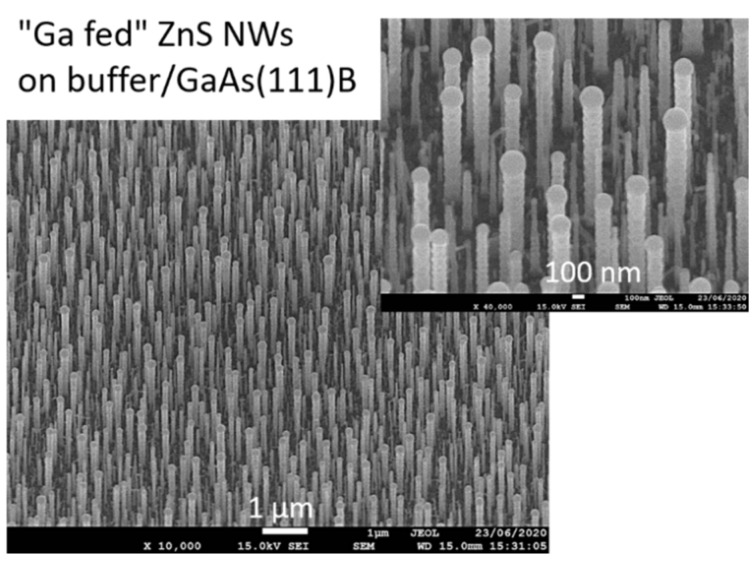
SEM images of ZnS NWs on ZnS (buffer)/GaAs (111)B, using “Ga-fed” Au-droplets.

We performed the following growth experiment. A GaAs (111)B substrate with ZnS buffer and 0.1 nm Au-deposition was introduced into the reactor. During the temperature elevation, between 450 and 500 °C, a 1 sccm TMGa flow (corresponding to a partial pressure of 4.5 Pa) was supplied to allow Ga diffusion into the Au droplets. The TMGa flow was stopped before a purge time of 30 s. Then, the dewetting was done at 500 °C for 10 min. The DEZn and DTBS flows were sent afterward to initiate the nanowire growth. Zn and S partial pressures were 2 and 4 Pa, respectively, corresponding to low growth rate conditions (see Table 1, NW15, and Figure 4a). A SEM image of the sample is given in Figure 14. We found very particular NWs, vertically well-aligned, exhibiting a big droplet on their top, and quasi-periodic micro-facets on their lateral surfaces.

**Figure 15:**
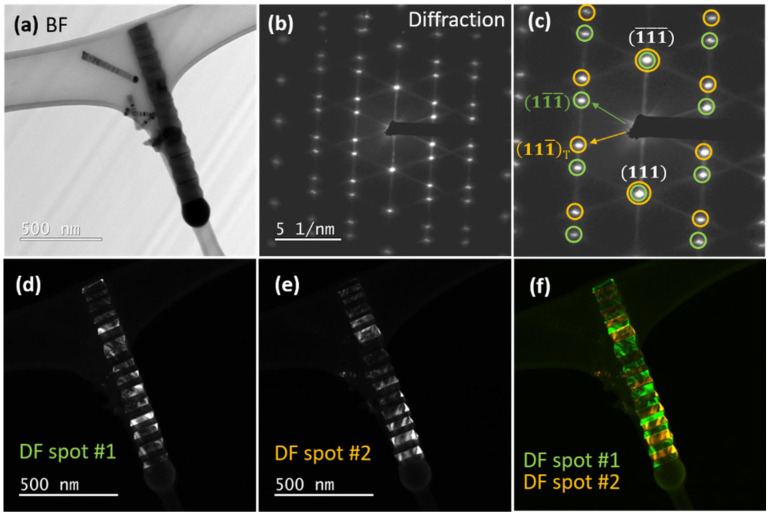
(**a**) bright-field TEM image of a single NW; (**b**) selective area electron diffraction pattern of the NW indicating a ZB twinning symmetry, (**c**) marked diffraction twinning spots with green and orange circles; (**d**) dark field (DF) image with spot #1 (green) highlighting segments from one ZB orientation; (**e**) DF image created from spot #2 (orange) highlighting segments from the other ZB orientation; (**f**) composite DF image made of ZB segments with two different orientations marked in green and orange.

To go further, we performed a preliminary structural analysis using TEM. The bright field-image of a single NW is shown in Figure 15a. The catalyst droplet can be seen at the top of the NW. The diameter of the NW is around 140 nm and a zigzag faceted structure can be seen on the sidewalls. The related SAED pattern is given in Figure 15b. The diffraction pattern of the whole NW shows a symmetry that suggests the presence of twinning. The NW would be periodically twinned, made of an alternating structure involving the two possible orientations of ZB structure (refer to Figure 10). To confirm that, we selected two different spots related to each orientation (see orange and green circles in Figure 15c), and performed dark field (DF) images. These DF images are shown in Figure 15d,e. It is clear that the contrast comes from different orientations. Indeed, if we select the diffraction spot #1 (in green) we see bright segments related to this orientation in Figure 15d, and, on the other hand, the selection of spot #2 (in orange) gives bright zones corresponding to the other orientation (Figure 15e). In Figure 15f, the composite image alternates green and orange segments and clearly illustrates the zig-zag structure. The period is here ~40 nm.

In recent years, the twinning superlattices, i.e., the formation of periodic rotational twins, have been introduced intentionally, or unknowingly in a number of III-V (InP [49], InAs [50], and GaAs [51]), II-VI (ZnS [20], ZnSe [52]), and IV-IV (SiC [53]) semiconductor nanowires. Apart from providing fundamental insight into the growth process, periodic twinning was anticipated for bandgap engineering and the generation of direct intersubband optical transitions, increased mechanical strength, and phonon engineering. A twinning superlattice is predicted to induce a direct bandgap in semiconductors that are normally indirect, such as silicon and gallium phosphide. However, controlling and maintaining periodic rotational twins in the NW structure is challenging.

Recent studies on III-V semiconductor nanowires have revealed periodic zinc-blende twins, which are typically created as a result of a high dopant concentration (Zn, Te, or Be) in the nanowire structure [49,51,54]. For example, Algra et al. commonly observed WZ phase in undoped InP NWs grown by MOCVD, but the addition of DEZn in the gas phase, used for p-type doping, resulted in the formation of a ZB crystal structure which was periodically twinned [49]. On the base of a nucleation model, they argued for a strong relation between the Zn atoms and the InP growth interface, leading to a decrease of the liquid-solid (LS) step energy for a ZB nucleus, compared to the WZ case. Interestingly, they demonstrated how the cross-sectional shape of the growing NW, constantly evolving from hexagonal to triangular and vice versa due to (111)A and (111)B side facets inclined in an opposite way, induces the periodic distortion of the catalyst droplet (to be relaxed via a twin creation), and hence explains the formation of a twinning superlattice.

However, some experimental works produced TSL without dopant [50,53]. For instance, in InAs NWs, Caroff et al. showed a dependence of the crystal structure on diameter, as well as on growth temperature. For small nanowires (<40 nm) they reported WZ structure with appearance of few stacking faults. With further increase in the diameter, they observed an increase of the stacking faults density, and inclusions of small ZB segments. For large diameters (>100 nm) they found out InAs coherent twin-plane superlattices.

In our case of ZnS 1D nanostructures, TSL are achieved by increasing the Ga composition of the droplet. We reported that Au_2_-Ga catalyst particles promote the VLS formation of the 15R crystal phase ([17], NWs described in Section 5.1), which means 33% Ga, corresponding to the eutectic composition. Considering the large size of Ga fed droplets, we expect their Ga content to be much higher. Such difference in the droplet composition would have a significant effect on the droplet contact angle, interface energy between droplet and NW (liquid-solid, LS), and interface energy between droplet and vapor (liquid-vapor, LV). In the reported nucleation models [46], this variation of the catalyst composition has a strong impact and may favor the formation of ZB polytypes. Then, if we assume that (111)A and (111)B side facets are favored and kept as long as the distortion of the Ga-rich droplet allows it, a periodically twinned ZB structure would grow following the mechanism described above for III-Vs.

ZnS TSL have also been reported without additional doping. As mentioned in the introduction, Hao et al. [20] achieved ZB periodic structures, however with a very short period of a few nanometers. To the best of our knowledge, our ZnS TSL are the first achieved ones with a large period of 40 nm. Actually, achieving ZB phase perfection in ZnS NWs is pretty rare in the literature, and we discussed in the previous section that the small stacking fault energy would favor hexagonal phase or other polytypes. The scarcity of long pure ZB segments in ZnS NWs explains itself the difficulties to achieve it. Interestingly, our results are in agreement with the work of Liang et al. [23]. They used pure Ga as a catalyst on GaAs (111)B substrates to obtain vertical arrays of ZnS NWs (exhibiting diameters > 100 nm) and reached a ZB structure with frequent inclusions of stacking faults, nevertheless not showing periodicity.

## 6. Conclusions

In conclusion, a comprehensive investigation has been conducted on the catalyzed growth of ZnS nanowires by using MOCVD. After conducting a preliminary study on thin film growth to identify the different MOCVD growth regimes, it was found that the nanowires emerge in the re-evaporation regime (550 °C) and under sulfur-rich conditions (S/Zn ratio of 2). GaAs (111)B substrates are confirmed to give the best arrays of NWs, in terms of verticality and uniformity, in agreement with the literature. From TEM experiments on single NWs, we identified the occurrence of different polytypes, depending on the growth conditions. Based on our previous work, we recalled that catalyzed growth in the VSS conditions involving a pure solid Au-droplet leads to a mixed WZ (dominant)-ZB structure, while in VLS conditions (liquid Au-Ga droplet), an original 15R high order polytype is observed. From that starting point, the partial pressure of precursor was varied in the MOCVD reactor, and supersaturation is demonstrated to play an important role on the crystal phase selection, as it transforms the structure from 15R (high order polytype) to 2H (hexagonal phase). The growth mechanisms are discussed and understood on the light of published works on III-V NWs, and our experimental findings agree with theoretical models. Hence, it is suggested that further calculations taking into account the specific properties of ZnS, such as its low stacking fault energy, would be worth performing. Finally, another remarkable structure, a twinning superlattice made of zinc-blende segments of a few tens of nanometers sandwiched between twin boundaries, was achieved by increasing the Ga composition of the Au-Ga alloy catalyst. Indeed, the richness and the variety of the crystal phases attainable in ZnS, as well as the easy fabrication in the nanostructured form, make this material particularly interesting for the study and the general comprehension of polytypism in semiconductor nanowires.

## Figures and Tables

**Table 1:** Sample list, indicating growth conditions.

	Study	Ref. of Sample	Tg (°C)	P_DEZn_ (Pa)	P_DTBS_ (Pa)	VI/II Ratio	Substrate
Thin films		TF1	320		15.6	2	
		TF2	360		15.6	2	
		TF3	390		15.6	2	
		TF4	420		15.6	2	
	Temperature	TF5	450		15.6	2	
		TF6	480		15.6	2	
		TF7	510	7.8	15.6	2	GaAs (100)
		TF8	540		15.6	2	
	_____	TF9	570		15.6	2	
		TF10	450		5.1	0.6	
		TF11	450		7.8	1	
	VI/II ratio	TF11	450		11.7	1.5	
		TF13	450		15.6	2	
		TF14	450		25	3.2	
Nanowires		NW1	450		15.6	2	
		NW2	500		15.6	2	
	Temperature	NW3	525		15.6	2	
		NW4	550		15.6	2	
	_____	NW5	575	7.8	15.6	2	GaAs (111)B
		NW6	550		5.1	0.6	
	VI/II ratio	NW7	550		7.8	1	
		NW8	550		11.7	1.5	
	_____	NW9	550		25	3.2	
	Substrate	NW10	550		15.6	2	GaAs (100)
							and ZnS buffer
	Substrate	NW11	550		15.6	2	GaAs (111)A
							and ZnS buffer
	Substrate	NW12	550		15.6	2	GaP (111)
							and ZnS buffer
	High V_g_	NW13	550	12	24	2	GaAs (111)B
	Low V_g_	NW14	550	2	4	2	GaAs (111)B
Ga feeding nanowires	TMGa	NW15	550	2	4	2	ZnS buffer
	4.5 Pa						
